# Innovative Materials as Micronutrient Carriers in Soybean Cultivation

**DOI:** 10.3390/ma18092070

**Published:** 2025-04-30

**Authors:** Marzena S. Brodowska, Mirosław Wyszkowski, Ryszard Grzesik

**Affiliations:** 1 Department of Agricultural and Environmental Chemistry, University of Life Sciences in Lublin, Akademicka 15 Str., 20-950 Lublin, Poland; 2 Department of Agricultural and Environmental Chemistry, University of Warmia and Mazury in Olsztyn, Łódzki 4 Sq., 10-727 Olsztyn, Poland; 3 Jednostka Biznesowa Agro, Grupa Azoty Zakłady Azotowe Kędzierzyn S.A., Mostowa 30 A Str., 47-220 Kędzierzyn-Koźle, Poland; ryszard.grzesik@grupaazoty.com

**Keywords:** innovative chelates, soybean, SPAD, biometrics, yield, elements, soil properties

## Abstract

Many of today’s innovative materials used to carry trace elements (TEs) are derived from chelates. Most of the materials used for this purpose have been produced on the basis of EDTA, which is not considered to be environmentally friendly due to its high persistence. Research is therefore being carried out to produce materials that do not pose an environmental risk. Therefore, a study was carried out to determine the effects of newly developed innovative materials with embedded biodegradable and environmentally safe chelates (IDHA—iminodisuccinic acid—and N-butyl-D-gluconamide ligands) containing copper, molybdenum and iron on the yield, biometric characteristics and chemical composition of soybean and selected soil properties. It is difficult to find publications on their effects in soybean cultivation. The greatest increase in soybean leaf greenness index (SPAD) was found after the addition of pure Salmag^®^ (Sal.^®^). The effect of the chelates on the SPAD index was lower, with Sal.^®^ + Fe chelate having the greatest effect during the vegetative development stage and Cu chelate having the greatest effect during the flowering stage. Sal.^®^ + Cu, especially with Fe, accelerated pod and seed ripening in the last vegetative stage of soybean. Sal.^®^ + Cu had the most favourable impact on plant height, pure Sal.^®^ on the pod number per plant, Sal.^®^ + Fe on the seed number per pod, Sal.^®^ with Mo and Fe chelates on soybean seed yield, and pure Sal.^®^ on fresh weight remaining above-ground part yield, while pure Sal.^®^ and Sal.^®^ + Fe had the most favourable impact on dry weight aerial yield. The fertiliser materials (especially Sal.^®^ + Cu) generally increased the N content of the tested soybean organs and the Cu content of the other above-ground soybean parts (especially those containing chelates) and had an antagonistic effect on the Mg content of the soybean above-ground parts. Sal.^®^ + Cu also had a negative effect on the Fe content of other above-ground soybean parts. Sal.^®^ + Fe had a positive impact on the iron content, and Sal.^®^ + Mo had a positive impact on the molybdenum content of soybean. The applied fertilisers had little effect on the contents of Cu, Mo and Fe in the soil. There was only a significant increase in the Cu content of the soil after the addition of Sal.^®^ + Cu and a significantly smaller increase under the influence of Sal.^®^ without chelates, as well as an increase in the Mo content of the soil with Sal.^®^. The present study confirms the beneficial impact of the novel materials with chelates. It has been demonstrated that the presence of materials containing Mo and, in particular, Cu has a considerable effect on the yield and quality characteristics of soybeans.

## 1. Introduction

Many innovative materials used today to carry trace elements (TEs) are derived from chelates [1]. Chelates are formed by combining a chelating compound (ligand) with a metal cation. The most commonly used chelating compounds are EDTA (ethylenediaminetetraacetic acid), DTPA (diethylentriaminepentaacetic acid), EDDHA (ethylenediaminodihydroxyphenylacetic acid), HEEDTA (hydroxyethylenediamino-triacetic acid), IDHA (iminodihydroxyphenylacetic acid) and HBED (hydroxybenzyl-ethylenediaminetetraacetic acid). In contrast, the most common TE cations used to form chelates are zinc (Zn), copper (Cu), manganese (Mn) and iron (Fe) [2,3]. Most of the materials used for this purpose have been produced on the basis of EDTA, which is not considered to be environmentally friendly due to its high persistence. Research is therefore being carried out to produce materials that do not pose an environmental risk.

Chelates are widely used, most commonly in medicine in chelation therapy (mainly EDTA) for the treatment of heavy metal poisoning or cardiovascular diseases (e.g., atherosclerosis); in biochemistry to stabilise enzymes and proteins (protecting them from degradation and increasing their activity); and in agriculture to increase the availability of micronutrients for plants, which is crucial for proper plant growth and development [4].

Chelates are increasingly being used in agriculture as an alternative and more efficient method of supplying essential nutrients, especially TEs [5]. Synthetic chelates such as EDTA, DTPA, HBED and IDHA are used in agriculture for soil fertilisation, but most of the preparations produced to date are based on EDTA [4]. Their foliar application gives particularly good results, allowing direct and, above all, rapid delivery of micronutrients to plants, especially during periods of high nutrient demand at key stages of plant growth or under stress conditions such as drought and flooding. It is possible to deliver them rapidly as an intervention together with chelate-based materials used to improve soil fertility [6]. The structural characteristics of chelates impede the amalgamation of micronutrient ions with components inherent in the soil solution, thereby facilitating their absorption by plant leaves. The sequential distribution of micronutrients has been demonstrated to enhance their absorption by plants and mitigate the risk of overaccumulation [7]. They then become effective in preventing micronutrient deficiencies, leading to the proper growth of better and higher-yielding plants. Chelates applied to the foliage act more quickly than those applied to the soil, but their effect is usually shorter (from a few days to even a few weeks).

Some chelates (e.g., EDTA, DTPA and EDDHA) are quite persistent in soil, and their duration of action varies from a few weeks to even a few months. Nevertheless, the efficacy of these substances is contingent upon the characteristics of the soil. They demonstrate optimal performance in soils with a pH close to neutral, while their effectiveness is significantly diminished in soils with a pH that is excessively high or low. An example is EDTA chelates, which are less effective in high-pH soils because they become less stable [4]. The opposite is true for EDDHA chelates, which are more stable [3]. According to Klem-Marciniak et al. [4], EDDHA and ethylenediamino-N,N′-di[(2-hydroxy-5-sulphophenyl)acetic acid (EDDHSA) chelates give good results in supplementing deficiencies of certain TEs (e.g., iron) in all soil pH ranges. Chelates of individual TEs can be used together with chelates of other metals or with other materials used for soil fertilisation. However, care must be taken to ensure that these chelates are compatible with each other and with other fertilisers used to grow individual crop species. Some chelates may not be fully utilised by plants because of unfavourable reactions with each other or with other fertiliser components. The dosage of chelates should also be adapted to the requirements of the individual plant species and other growing conditions. Combining different chelates can help optimise micronutrient availability and minimise the risk of environmental contamination [8].

All materials used in agriculture should be safe for the environment. Therefore, the durability of a given material or preparation is an important factor. It is important that it has a high level of biodegradability, as this will rapidly reduce its harmfulness to the environment. The chelates used to date have varying degrees of biodegradability. Chelates with an EDTA carrier are among those that are less biodegradable and remain in the environment longer than, for example, IDHA-based chelates [3]. Poorly biodegradable chelates can leach into groundwater, changing its chemical composition and causing contamination. This can also affect the quality of water used for crop irrigation [9]. The use of IDHA-based chelates reduces the risk of environmental contamination, as they degrade more rapidly in soil [8].

The use of chelates confers numerous benefits in comparison with conventional mineral fertilisers. Because chelates move more readily across plant cell membranes, resulting in rapid and efficient transport of micronutrients to individual plant organs, they are more readily absorbed by plants than conventional mineral fertilisers. Chelates are more stable than typical mineral fertilisers under varying soil conditions—high or low pH [10]. Some components of typical mineral fertilisers can form insoluble complexes in the soil, limiting the bioavailability of TEs to plants. The structure of chelates also allows easy migration of TE ions in soil solutions [8]. Chelates act more rapidly than conventional mineral fertilisers, especially when applied as foliar sprays, which is particularly important during critical stages of plant growth when the demand for individual elements is particularly high. Better utilisation of the components from chelates makes it possible to reduce their doses for cultivated plant species [11]. The good water solubility, relatively low dissociation and slow release of TEs of chelates means that plant uptake of these nutrients is high but not excessive. Chelates biodegrade faster than mineral fertilisers and therefore have a less negative impact on soil and the environment [7]. They are therefore more environmentally friendly.

It is important to find a suitable material that is biodegradable and environmentally friendly while at the same time ensuring that plants are supplied with adequate amounts of nutrients [12]. A study was conducted to ascertain the effects of introducing TEs into soil using new innovative IDHA-based chelates and to test their effects on plants. Soybean (*Glycine* Willd.) was used as the test plant. It is difficult to find publications on IDHA effects in soybean cultivation.

Soybean is a staple crop that plays a key role in the global food and feed economy. It is predominantly cultivated in the USA, Brazil and Argentina. It is one of the main sources of vegetable protein and is used as a basic ingredient and raw material providing essential amino acids in the production of animal feed (for poultry, pigs and cattle). It is also used in the food industry (to produce soya milk, tofu, soy sauce and soya oil). Soybean oil is also used in other industries, including the production of biofuels, cosmetics and paints. Soybean is of ecological significance due to its capacity to fix atmospheric nitrogen. This process has a beneficial effect on soil structure and reduces the reliance on nitrogen fertilisers [13,14].

With the above factors in mind, a study was carried out to determine the effects of new innovative materials with embedded biodegradable and environmentally safe chelates (IDHA—iminodisuccinic acid—and N-butyl-D-gluconamide ligands) containing copper, molybdenum and iron on plants and soil. The innovative chelate-based materials containing the above trace elements were compared with a commercial solid fertiliser (Salmag^®^). The following research hypotheses were formulated: (1) The new innovative Salmag^®^ solid fertiliser-based materials with chelates containing Cu, Mo and Fe would improve soybean biometric traits, such as plant height, number of pods per plant and seed weight, and increase soybean yield. (2) The use of materials with chelates would increase the contents of macronutrients and micronutrients in seeds and other above-ground parts of soybeans compared to traditional fertilisers. (3) Innovative materials containing Cu, Mo and Fe chelates would positively influence selected soil properties, such as pH and contents of selected macronutrients and trace elements.

## 2. Materials and Methods

### 2.1. Methodological Considerations

An experimental pot study was conducted in the vegetation hall of the University of Warmia and Mazury in Olsztyn (Poland). The research was carried out in 9 kg pots filled with soil material from the arable layer of a typical eutrophic brown soil, which, according to the classification of soils [15], belongs to the loamy sands. The granulometric composition and basic characteristics of the soil are outlined in Table 1. The effects of the new innovative materials with applied chelates containing copper (Cu), molybdenum (Mo) and iron (Fe) was compared with that of classical mineral fertiliser Salmag^®^ (salpetre—ammonium nitrate with calcium carbonate and magnesium carbonate mixture) in relation to a control object, where no nitrogen was applied. The composition of Salmag^®^ (Sal.^®^) included nitrogen—27% N, calcium—5.0% CaO, and magnesium—2.4% MgO [16]. The newly developed innovative chelate-containing materials were produced in a series of trials specifically for the research conducted by Grupa Azoty Zakłady Azotowe Kędzierzyn S.A. (Kędzierzyn-Koźle, Poland). They were produced on the basis of Sal.^®^ encapsulated with chelates of Cu, Mo or Fe. Their trace element contents were as follows: Sal.^®^ + Cu chelate—0.25% Cu, Sal.^®^ + Mo chelate—0.25% Mo, and Sal.^®^ + Fe chelate—0.25% Fe. IDHA chelates and biodegradable particles were used to produce the innovative materials. During their manufacture, innovative chelate coatings containing TEs (Cu, Mo and Fe) were added to cooled Sal.^®^ granules in a fluidised bed. Laboratory-produced IDHA chelates of Fe and Mo were used, produced in the laboratory using a new method for the production of iminodisuccinic acid (IDHA—C_8_H_11_O_8_N), while the innovative patented [17] ligand N-butyl-D-gluconamide was used to produce the Cu chelate. All chelates used were characterised by laboratory-confirmed biodegradability. The test crop was soybean (*Glycine* Willd.) of the Ambella variety. Equal initial amounts of nitrogen—30 mg kg^−1^ of soil, phosphorus—60 mg kg^−1^ of soil (Super Fos Dar 40, 41.2% P_2_O_5_), and potassium—80 mg kg^−1^ of soil (potassium salt, 60% K_2_O) were applied to all the experimental sites. Innovative materials containing chelates and P and K fertilisers were mixed with the soil and poured into pots according to the experimental design. Soybean seeds (6 plants per pot) were sown in the prepared soil. The research was conducted in five replicates, the moisture content of the soil being constant at 60% of the water capacity of the field. The soybeans were harvested at technical maturity (after pod and seed maturity, BBCH 89). At harvest, the height of the plants was measured, and the yield of seeds and other above-ground parts (straw), the 1000-seed mass (TSM), and plant and soil samples were collected for laboratory analysis.

### 2.2. Laboratory and Statistical Analysis Methods

The leaf greenness index (Soil and Plant Analysis Development, SPAD) was determined during vegetative development (BBCH14), flowering (BBCH65), and pod and seed maturation (BBCH89). SPAD determinations were performed using a Konica Minolta Optics SPAD-502Plus instrument (Konica Minolta, Inc., Chiyoda, Japan). Plant height was measured with a tape measure, and plant yield and 1000-seed mass were determined using the weight method. The contents of macronutrients (N, Ca and Mg) and TEs (Cu, Mo and Fe), which were components of the innovative chelate-containing materials and could affect their levels in the plants, were analysed in the harvested and dried plants. The determination of macronutrient and TE contents in the plants was carried out by the following methods: N contents were determined by the Kjeldahl method [18]; Ca and Mg contents were determined by the atomic absorption spectrometry method (AAS) [19]; and Cu, Mo and Fe contents were determined by the AAS method [19]. Samples of plant material for macronutrient analysis were digested in concentrated H_2_SO_4_.

Analysis of the contents of the above-mentioned macronutrients and TEs in the soil used to set up the experiment and after the soybean harvest was also performed. The methods used for soil analysis were as follows: TOC contents were determined with the TOC Analyzer (Shimadzu Corporation, Kyoto, Japan) using the Solid Sample Module (SSM-5000A); N contents were determined by the Kjeldahl method [18]; P contents were determined by the colorimetric method [19]; K, Ca, Mg, Cu, Mo and Fe contents were determined by the ASA method [19]. Soil for N determinations was digested in concentrated H_2_SO_4_ [18], and for the Ca, Mg, Cu, Mo and Fe analyses it was digested in royal water [19]. In addition, the pH in 1 M KCl was determined in the soil using the potentiometric method [20], and soil granulometric composition was also determined in the initial soil using the laser diffraction method [21]. The NCS ZC 73030 CRM and Fluka standard reference materials were used for the laboratory analyses.

The results were subjected to statistical verification using one-way ANOVA at a significance level of *p* ≤ 0.01. In addition, principal component analysis (PCA) was employed to ascertain the relationships between individual parameters. The STATISTICA 13.3 program [22] was used for statistical calculations.

## 3. Results

### 3.1. Plants

#### 3.1.1. SPAD Index

**Figure 1:**
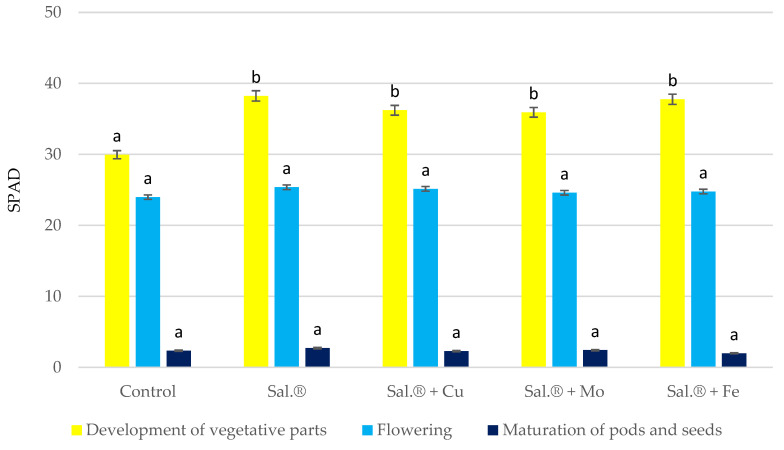
Soybean SPAD index in stages: stem shooting, flowering, and maturation of pods and seeds. Sal.^®^—Salmag^®^; the different letters above the bars attest to a significant influence at *p* ≤ 0.01.

The soybean SPAD index was highest during vegetative development and decreased as the growing season progressed (Figure 1). The leaf greenness index of soybean ranged from 29.96 (control) to 38.24 (Sal.*^®^*) at the vegetative part development stage, from 23.99 (control) to 25.38 (Sal.*^®^*) at the flowering stage, and from 1.99 (Sal.*^®^* + Fe) to 2.72 (Sal.*^®^*) at the pod and seed maturation stage. During the vegetative part development and flowering stages, each of the fertilisers applied to the soil increased the greenness of the leaves. In the last stage of soybean vegetation, their effect was less clear. The greatest increase in soybean SPAD index values was found after the application of Sal.*^®^* without micronutrients. In comparison with the control (without N), this was 28% at the vegetative part development stage, 6% at the flowering stage, and 16% at the pod and seed maturation stage.

The effect of the chelate additives on the SPAD index was smaller, with Sal.*^®^* + Fe chelate exhibiting the most significant effect at the vegetative part development stage and Sal.*^®^* + Mo chelate displaying the least significant effect during the flowering stage with Cu chelate and Mo chelate, respectively. Sal.*^®^* with Mo chelate also contributed to a slight (3%) increase in the SPAD index at pod and seed maturity. Sal.*^®^* + Cu chelate and especially Sal.*^®^* + Fe chelate accelerated pod and seed ripening in the last stage of soybean vegetation. This was shown to result in a decline in the SPAD index of this crop in comparison to the control.

#### 3.1.2. Plant Biometric Properties

**Figure 2:**
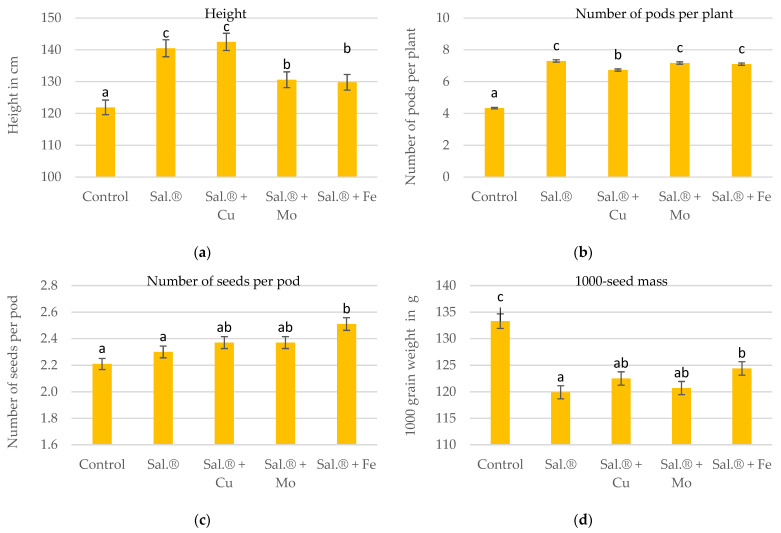
(**a**) Height at full maturity (in cm), (**b**) number of pods per plant, (**c**) number of seeds per pod and (**d**) 1000-seed mass (in g) of soybean. Sal.^®^—Salmag^®^; the different letters above the bars attest to the significant influence at *p* ≤ 0.01.

The results demonstrate a clear correlation between the application of fertiliser and the parameters of plant height (Figure 2a), the number of pods per plant (Figure 2b) and the number of seeds per pod (Figure 2c). The Sal.*^®^* + Cu chelate exhibited the most favourable outcomes in terms of plant height, while pure Sal.*^®^* demonstrated the most significant impact on the number of pods per plant and Sal.*^®^* + Fe chelate enhanced the number of seeds per pod, increasing them by 17, 69 and 14%, respectively, in comparison to the control. The Fe chelate exhibited the least significant positive effect on plant height, while the Cu chelate demonstrated the most marked effect on the number of pods per plant. Pure Sal.*^®^* exhibited the most pronounced effect on the number of seeds per pod.

None of the applied materials had a positive effect in terms of increasing the 1000-seed mass of the plants, and the changes observed for the individual micronutrient supplement fertilisers were relatively small (Figure 2d). As a result of the application of fertilisers with micronutrient additives, the decrease in the weight of 1000 soybean seeds varied between 7 and 10% in relation to the control object.

#### 3.1.3. Crop Yield

The lowest soybean yields (fresh and dry matter) were found in the control (Table 2). Sal.*^®^* without chelates increased the soybean fresh matter yield by 79% and the dry matter yield by 103% in comparison with the no-N object. The yield-enhancing effects of Sal.*^®^* + Mo chelate and Sal.*^®^* + Fe chelate on soybean seeds was higher, and the effect of Sal.*^®^* + Cu chelate was lower than that of pure Sal.*^®^*. Under the influence of Sal.*^®^* + Fe chelate, soybean fresh matter yield was increased by 95%. Furthermore, the impact on soybean dry matter yield was particularly pronounced, exhibiting a 119% increase compared to the control.

As demonstrated in Table 1, the application of fertilisers resulted in a significant enhancement of the yield of the other above-ground parts of the soybean. However, this effect was considerably less pronounced in comparison to the impact observed on the seeds. Under their influence, the increase in the yield of soybean above-ground parts ranged from 21% (Sal.*^®^* + Mo) to 26% (Sal.*^®^* without chelates) for the fresh weight and from 40% (Sal.*^®^* + Cu) to 44% (Sal.*^®^* without chelates and Sal.*^®^* + Fe) for the dry weight yield compared to the control (without N).

#### 3.1.4. Macroelements

Sal.*^®^* exerted no significant effect on seed nitrogen (N) levels, yet it induced an 11% increase in N levels in the above-ground parts of soybean (Table 2). All micronutrient enriched chelates had a stronger effect than Sal.*^®^*, causing an increase in the N content in soybean seeds of 6% (Sal.*^®^* + Mo), 8% (Sal.*^®^* + Fe) and 12% (Sal.*^®^* + Cu). The applied fertiliser had no clear effect on the Ca content of the seeds, with maximum differences not exceeding 4%. Sal.*^®^* + Mo and Fe chelates caused a small but significant reduction in the Mg content in soybean seeds compared to the control.

Sal.*^®^* without micronutrient additives induced an 11% reduction in N and Ca contents in the remaining above-ground parts of the soybean in comparison with the control (Table 1). The positive effect of Sal.*^®^* + Cu in terms of increasing the concentration of total N in the above-ground parts was higher (16%), and the effect of Sal.*^®^* + Fe (11%) was comparable to that of pure Sal.*^®^* (11%). Sal.^*®*^ + Mo had no significant effect on the N in the above-ground parts of the plants. The Fe-enriched Sal.*^®^* promoted Ca accumulation in the seeds and above-ground parts of the soybean, resulting in a slight increase of 4% compared to the N-free object. The experiment revealed a stronger limiting effect on the Mg content of the soybean above-ground parts than in the seeds. Sal.*^®^* + Cu induced slightly greater changes, and Sal.*^®^* enriched with Mo and Fe induced smaller changes than pure Sal.*^®^*. Compared to the N-free control, the reductions in Mg contents were 11% (Sal.*^®^* + Cu), 10% (Sal.*^®^* and Sal.*^®^* + Mo) and 5% (Sal.*^®^* + Fe).

#### 3.1.5. Microelements

The used chelated materials were demonstrated to exert a significant influence on the micronutrient composition of the plants (Table 2). The Cu content of soybean seeds in the fertilised objects exhibited no significant difference from that in the control object; however, the addition of Cu chelate to Sal.*^®^* resulted in a slight increase in the Cu content of soybean seeds. A positive effect of fertilisation on Mo and Fe contents in soybean seeds was found only after the addition of Sal.*^®^* + Fe and Sal.*^®^* + Mo. Sal.*^®^* + Fe and Sal.*^®^* + Mo increased the Mo content by 5 and 11%, respectively, and the Fe content by 8 and 4% compared to the control.

Greater changes in Cu content were observed in the above-ground parts of the soybean than in the seeds. The chelates had a positive effect on the Cu content in the above-ground parts of the soybean plants, causing an increase of 9% (Sal.*^®^* + Mo), 12% (Sal.*^®^* + Fe) and 13% (Sal.*^®^* + Cu) in comparison with the object without N. A positive effect of chelates on the Mo content in soybean straw was only observed after soil enrichment with Sal.*^®^* + Mo. The impact of fertilisers on the Fe content of soybean above-ground parts was less evident. A 26% increase in the Fe content of soybean above-ground parts was observed after the addition of Sal.*^®^* + Fe. Sal.*^®^* and Sal.*^®^* + Cu chelate, on the other hand, contributed to a 31% and 32% reduction in Fe content in the remaining above-ground parts of the soybean.

### 3.2. Soil Properties

#### 3.2.1. pH

The pH of the soil was measured in the aftermath of the soybean harvest, and the results obtained ranged from 5.24 (Sal.*^®^* + Mo) to 5.45 (Sal.*^®^* + Fe) (Table 3). Most of the applied materials had no significant effect on soil pH. The exception was Sal.*^®^* + Mo chelate, which resulted in a slight decrease in soil pH (5.24) in comparison to the control (5.37).

#### 3.2.2. Macroelements

The impact of the fertilisers, both in their pure form and following the incorporation of Cu, Mo and Fe chelates, on the N and Mg contents of the soil was not significant (Table 3), although the N content was higher in the post-harvest soil of plants fertilised with Sal.*^®^* in conjunction with micronutrient additives when compared to the control, which did not contain N. This was particularly evident in the post-harvest soil of soybean fertilised with Sal.*^®^* + Fe. The impact of the fertilisers on the calcium content in soil was inconclusive. Pure Sal.*^®^* caused a slight decrease of 5% in the Ca concentration, while Sal.*^®^* + Mo caused a 6% increase in the level of this TE in the soil after the harvest of the test crop.

#### 3.2.3. Microelements

The application of fertilisers had a minimal impact on the concentrations of Cu, Mo and Fe in the soil, as evidenced in Table 3. A significant increase of 15% in the Cu content of the soil was observed following the application of Sal.*^®^* + Cu, while an 8% increase was recorded after the after application of pure Sal.*^®^* in comparison to the control. The application of Sal.*^®^* in the absence of micronutrient additives resulted in a 4% increase in the Mo content in comparison with the control series without N. The effect of the other fertilisers was found to be insignificant.

### 3.3. PCA Analysis

#### 3.3.1. Soybean Seeds

**Figure 3:**
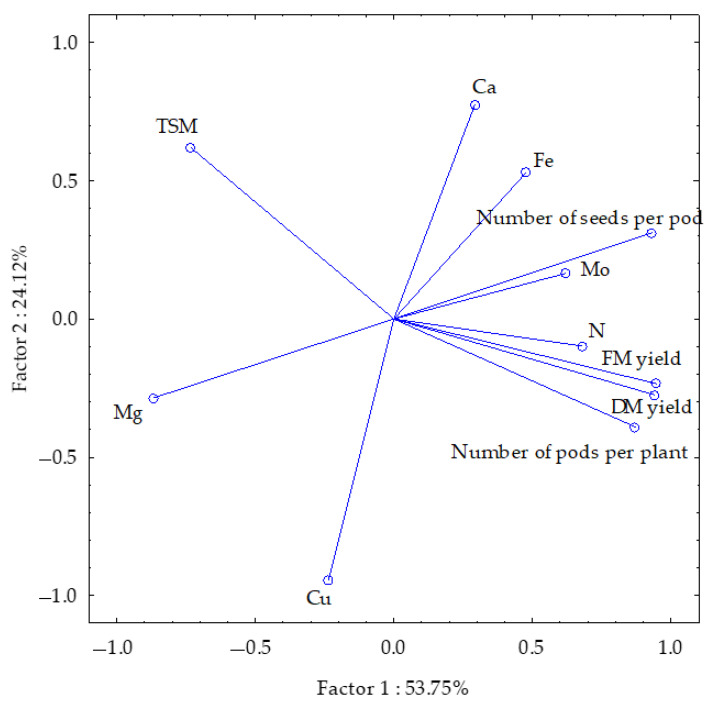
Yields and parameters of soybean seeds plotted using the PCA method. The following parameters are represented by the vectors: fresh and dry matter yield of seeds, 1000-seed mass (TSM), number of pods per plant, number of seeds per pod, and macro- and microelement contents.

The first group of parameters studied, including soybean fresh and dry weight yield, number of pods per plant and number of seeds per pod, and seed contents for Mg, Mo and N, were decisive in 53.75%, while the second group, including soybean 1000-seed weight and seed contents for Cu, Ca and Fe, were decisive in 24.12% of the total dataset correlation (Figure 3).

Most of the vectors, due to their similar lengths, were of similar importance with respect to the proportion of variation. Only the vectors reflecting N, Mo and Fe were shorter than the others. The strongest positive correlations were found between soybean fresh and dry matter yield and between seed yield and number of pods per plant (and less strongly with number of seeds per pod). The present study revealed an inverse correlation between the Mg content and the plant yield, as well as the number of seeds per pod. Furthermore, an inverse correlation was identified between the Ca content and the Cu content. Additionally, an inverse correlation was observed between the mass of 1000 seeds and the yield, as well as the number of pods per plant.

#### 3.3.2. Other Above-Ground Parts of Soybean

**Figure 4:**
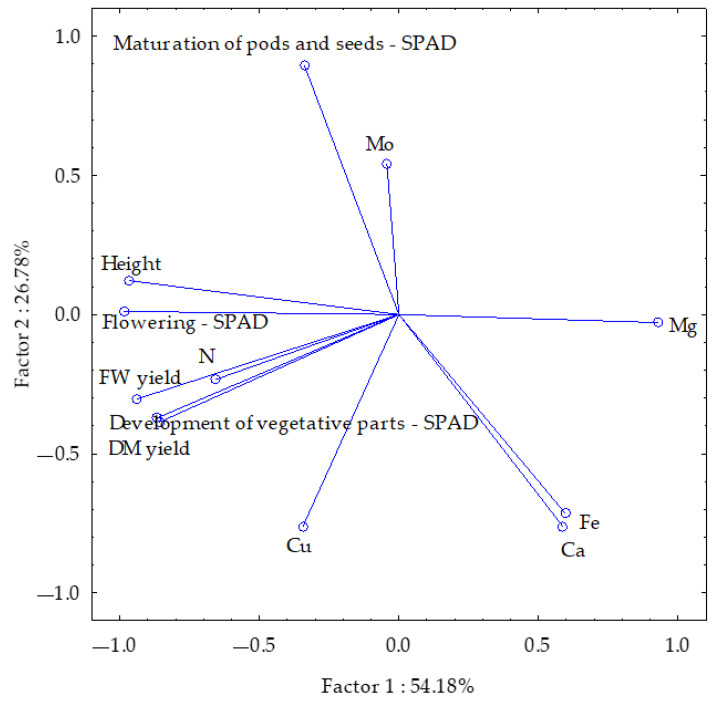
Yield and parameters of the other soybean above-ground parts examined plotted using the PCA method. The following parameters are represented by the vectors: SPAD index at development of vegetative parts, flowering, maturation of pods and seeds, plant height, fresh and dry matter yield of other above-ground parts, and macro- and microelement contents.

Group one included the soybean fresh and dry matter yield of above-ground parts, the SPAD index at the first two vegetative stages, plant height, and Mg content, and group two included the SPAD index at pod and seed maturity and Ca, Fe, Cu and Mo contents. These accounted for 54.18% and 26.78% of the total correlation, respectively (Figure 4). The length of the vectors was similar, with the exception of those reflecting N and Mo contents in the above-ground parts of the soybean. A strong positive correlation was identified between the fresh and dry weight yield of soybean above-ground parts and the SPAD index during the vegetative part development stage. A weaker correlation was observed between the SPAD index at the flowering stage and plant height, as well as between Ca and Fe contents. Plant height; SPAD index at the flowering and, to a lesser extent, at the vegetative part development stage; and fresh and dry matter yield of soybean above-ground parts were negatively correlated with Mg content, and plant height and SPAD index at the flowering stage and the pod and seed maturity stages were negatively correlated with Ca and Fe contents of soybean above-ground parts.

#### 3.3.3. Soil After Soybean Harvest

**Figure 5:**
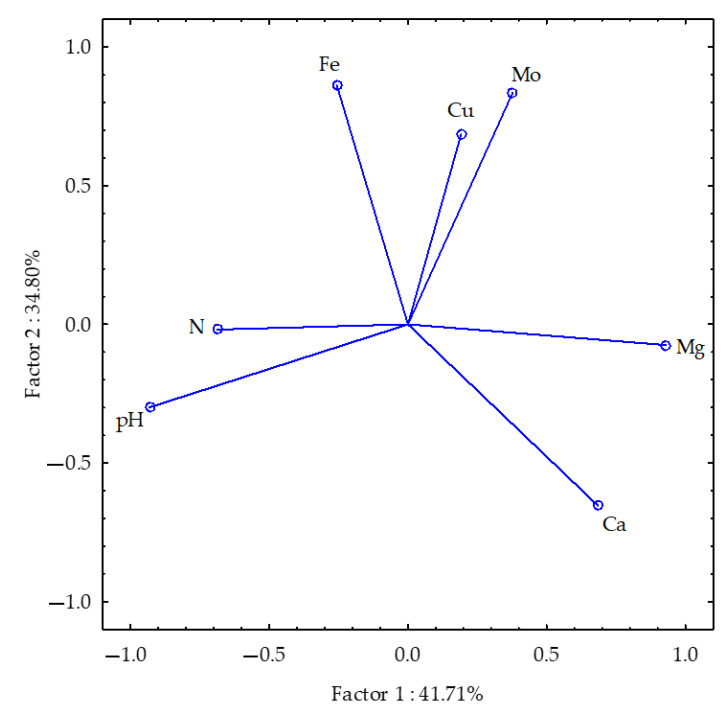
Selected post-harvest soil properties of soybean plotted using the PCA method. The following parameters are represented by the vectors: pH and soil macro- and microelement contents.

The total Mg and N contents and soil pH were found to be consistent with the first group, accounting for 41.71% of the total correlation of the dataset (Figure 5). In contrast, the soil Ca, Fe, Mo and Cu contents were found to be consistent with the second group, accounting for 34.80% of the total correlation of the dataset. The majority of the vectors (with the exception of soil N and Cu contents) exhibited comparable lengths, suggesting that they all contributed to the variability in a similar manner. The correlations between the studied parameters were relatively weak. Among the strongest were the positive correlation between Ca and Mg and the negative correlations between pH and Mg and Ca and between Ca and Fe.

## 4. Discussion

One potential application for innovative chelate-based materials is in agriculture, particularly crop production. In order to achieve maximum productivity and ensure the cultivation of specimens of the desired calibre, it is imperative that the requisite nutrients are supplied in sufficient quantities to facilitate the optimal growth and development of the plants. In addition to the classic macronutrients, TEs play a very important role, and their presence is indispensable for many enzymatic reactions [23]. Although their levels in plants are not necessarily high, they can be an important determinant of the correct rate of plant growth and development [24]. If they are not present in sufficient quantities in the soil, it becomes necessary to supply them to plants, which is a prerequisite for high crop yields. This is particularly important at critical stages of their development, especially at the beginning of the growing season and during fruit and seed formation. Interventions can then be made, although these should be carried out with fast-acting materials. These include some chelates used in combination with other fertiliser materials as well as micronutrients [11].

The chelates used in our study demonstrated a favourable impact on the growth and development of plants during vegetation, as evidenced by the acceleration of plant maturation, including soybean seeds, and their beneficial effect on biometric traits (SPAD, plant height, number of pods per plant and number of seeds per pod). The consequence of this was an increase in the yield of both seeds and other above-ground parts of the test plants. Chelates with Fe had the most beneficial effect, and in the case of seed yield, chelates with Mo also had a beneficial effect, as did chelates with Cu for biometric traits. This confirms research by other authors [25] which indicates that plant growth and planning can be reduced by up to 20% due to Cu deficiency, as this element is involved in photosynthesis and is an activator of many enzymes. This results in increased levels of proteins, carbohydrates, vitamins and other components in plants [26]. Iron is also involved in the same physiological processes in plants (photosynthesis, synthesis and regulation of enzymatic reactions or N metabolism, respiration, and redox reactions) [27]. Iron is generally abundant in soils, but its availability to plants can vary depending on soil properties [28]. Much of the iron is strongly bound to the soil [29], generally in the form of insoluble Fe^3+^, while plants take up Fe^2+^. This occurs mainly in well-oxygenated soils with a high pH [30]. In addition, a number of other factors, such as excessive soil contamination with various pollutants, including pesticide residues, and unfavourable metrological conditions can disrupt plant Fe uptake or alter their Fe requirements [27]. It is then necessary to supplement Fe, especially in the form of chelates, from which it can be better utilised than from traditional fertilisers. As demonstrated in the study by Xiao et al. [23], supplementation with Fe chelates resulted in increased chlorophyll content and accelerated photosynthesis. The study also revealed that there was an increase in the leaf and root number and in the above-ground part biomass and roots of the plants. In the experiment by Mahmoud et al. [5], the application of Fe chelates exerted a favourable influence on various plant characteristics, including height and leaf area, as well as fresh and dry plant weight. The study also demonstrated a positive impact on the number of shoots, number of pods and seeds, and seed weight. Chelates form complexes with micronutrients, making them more bioavailable to plants. This benefits fat synthesis in soybeans. Goli et al. [31] proved that chelates combined with iron ions (Fe) boost the oil content of soybean seeds. The oleic acid content soared from 13.0% to 33.5%, while that of linolenic acid dropped from 17.8% to 31.0%. This had a detrimental effect on the quality of the soybean oil, including its shelf life and oxidative stability. The role of Mo in plants is mainly related to the synthesis of enzymes (e.g., nitrate reductase) involved in N uptake and fixation [32], as well as the reduction of nitrate (N), which is then involved in protein and vitamin C synthesis and photosynthesis [26,33]. Application of Mo in the form of chelates and especially nano-Mo can result in a significant increase in plant biomass [34]. Molybdenum deficiency reduces Mo enzyme activity and N assimilation in the papillae of faba bean plants [35]. The effect of an adequate supply of micronutrients to plants is to produce a high yield of the desired quality.

The above-described effects of TE chelates on plant physiological processes were reflected in our own studies. An increase in the levels of Cu, Mo and Fe and of macronutrients, especially N, was found in the studied soybean organs. This confirms the results obtained by Brodowska et al. [12], who found analogous positive relationships between the addition of chelates (Cu, Mo and Fe) and their contents in the plant organs studied and between the application of Cu and Fe chelates and N accumulation, but only in straw. In contrast, Mahmoud et al. [5] showed an increase in the contents of some amino acids (arginine, isoleucine, phenylalanine, tyrosine and lysine) in the seeds of the studied plants. In addition, Muñoz-Márquez et al. [34] also observed a rise in the Mo content of plants following the application of a chelate of this element. As demonstrated by Mahmoud et al. [5], an increase in macronutrients (N, P, K, Ca and Na) and TEs (Fe, Zn, Mn and Cu) was observed in broad bean seeds. The negative effect of soil-applied materials on Mg content found in our study was due to the presence of N, which can act antagonistically to Mg accumulation in plants. The antagonism of N to Mg was also reflected in studies by other authors [36]. However, the antagonism between the two elements is not always obvious. The effect of nitrogenous fertilisers on Mg content depends on the plant species. An experiment by Surányi and Izsáki [36] showed an increase in N content in the above-ground parts of barley under the influence of N fertilisation, but no effect on Ca or Mg content was observed. Conversely, studies on spring wheat demonstrated an enhancement in Mg content in both grain and straw as a consequence of their interaction [12,37]. On the other hand, Cu, Mo and Zn chelates exhibited a comparatively diminished and weaker effect on the N and Mg contents of plants than conventional fertilisers. In a contrasting study, Xiao et al. [23] reported that supplementation with chelates resulted in significant increases in the macronutrient and TE contents of plants.

The application of classical mineral fertiliser-like materials has been demonstrated to exert an influence on soil properties, including pH, acidity, sorption capacity and even partial organic matter content, such that it is not inconsequential for plant uptake of individual elements [38,39]. However, this depends on the type and form of fertiliser, with nitrate fertilisers having a relatively weak effect on soil properties, as this element is only sorbed to a limited extent by the soil sorption complex [40]. Furthermore, in the case of chelates, this effect is even smaller. This has been confirmed in our own research, where, apart from an increase in the soil contents of the elements present in the chelates, there were virtually no major changes in other soil properties, as these did not exceed 6% and were usually insignificant. However, it should be remembered that the content of available forms of elements, especially micronutrients, is closely related to the soil response. Cu deficiencies in soils are usually found at neutral or alkaline pH [41], Fe deficiencies in alkaline soils [25], and Mo deficiencies in acidic soils, from which it is easily leached [42]. In the event of substantial quantities of organic matter or clay being present in these soils, there is an increased propensity for binding of TEs, thereby reducing their availability to plants [43]. It is therefore important to regulate soil acidity to ensure high levels of nutrient availability to plants [44]. The use of Cu, Fe and Mo chelates has been demonstrated to result in a minor decline in soil pH [12]. The studies conducted by these authors have indicated a nascent tendency for an augmentation in the levels of the elements under scrutiny in plant material. However, a clear correlation between these levels in plant material and those in soil (with the exception of Cu) after crop harvest has not been demonstrated. This is probably related to their significant uptake by the cultivated plants.

Trace element supplementation in micronutrient-deficient soils is therefore becoming an indispensable factor in achieving high yields of plants of the desired quality. This has been confirmed by those who have carried it out. The incorporation of innovative Fe chelate-based materials into soil has been demonstrated to exert a particularly beneficial effect on both the yield and chemical composition of soybeans. In the case of soybean seed yield, chelates with Mo also had an analogous effect, while chelates with Cu also influenced biometric characteristics. Due to the different nutritional requirements of different plant species, further research is needed to include other plant species that are important for feed and food production.

Studies conducted under controlled conditions in pot experiments indicate significant potential for the used chelate materials. It seems important to extend the research with the results of experiments carried out on a larger scale under field conditions. This would take into account the influence of other factors, such as meteorological conditions, which, due to their variability from year to year, significantly affect plant growth and development and the availability and uptake of individual elements by plants. As a result, the yield and quality characteristics of the crop, as well as the characteristics of the soil, are significantly affected. The testing of materials, once completed with different plant species, will be used to produce new innovative chelate-based fertilisers by Grupa Azoty Zakłady Azotowe Kędzierzyn S.A. (Poland).

## 5. Conclusions

The greatest increase in soybean leaf greenness index (SPAD) was found after the application of pure Sal.^®^. The effect of the chelates on the SPAD index was lower, with Sal.^®^ + Fe chelate having the greatest effect during the vegetative development stage and Cu chelate having the greatest effect during the flowering stage. Sal.^®^ + Cu, especially with Fe, accelerated pod and seed ripening in the last vegetative stage of soybean. Sal.^®^ + Cu had the most favourable impact on plant height, pure Sal.^®^ on pods per plant number, Sal.^®^ + Fe on seeds per pod number, Sal.^®^ with Mo and Fe chelates on soybean seed yield, and pure Sal.^®^ on fresh-weight remaining above-ground part yield, while pure Sal.^®^ and Sal.^®^ + Fe had the most favourable impact on dry weight aerial yield.

The fertiliser materials (especially Sal.^®^ + Cu) generally increased the N contents of the tested soybean organs and the Cu contents of the other soybean above-ground parts (especially those containing chelates) and had an antagonistic effect on the Mg contents of the soybean above-ground parts. Sal.^®^ + Cu also had a negative effect on the Fe contents of other soybean above-ground parts. Sal.^®^ + Fe had a positive effect on the iron content and Sal.^®^ + Mo on the molybdenum content of soybean.

The applied fertilisers had little effect on the contents of Cu, Mo and Fe in the soil. There was only a significant increase in the Cu content of the soil after the addition of Sal.^®^ + Cu and a significantly smaller increase under the influence of Sal.^®^ without chelates, as well as in the Mo content of the soil with Sal.^®^.

The present study confirms the beneficial impact of the novel materials with chelates. It has been demonstrated that the presence of materials containing Mo and, in particular, Cu has a considerable effect on the yield and quality characteristics of soybean.

## 6. Recommendations and Future Actions

Given the different nutritional requirements of different plant species, further research is needed on the new innovative materials containing chelates of micronutrients used in the experiment, including other plant species important for feed and food production. The testing of the materials, once completed with different plant species, will be used to produce new, innovative chelate-based fertilisers.

## Figures and Tables

**Table 1:** Properties of initial soil.

Parameter	Content
Granulometric composition (%)	
Sand (2.0–0.05 mm)	77.55
Dust (0.05–0.002 mm)	19.95
Clay (<0.002 mm)	2.50
pH value in 1 M KCl dm^−3^	5.85
Total macroelements (g kg^−1^ DM):	
Total organic carbon (TOC)	3.104
N	1.120
Ca	5.481
Mg	2.229
Available macroelements (mg kg^−1^ DM):	
P	22.16
K	145.0
Total microelements (mg kg^−1^ DM):	
Cu	7.829
Mo	0.959
Fe	10,108

DM—dry matter.

**Table 2:** Impact of innovative materials with chelates on soybean.

Fertiliser	Yield FM	Yield DM	N	Ca	Mg	Cu	Mo	Fe
	g pot^−1^		Content in g kg^−1^ DM			Content in mg kg^−1^ DM		
Seeds								
Control	5.35 ± 0.12 a	3.77 ± 0.08 a	33.68 ± 0.39 a	0.483 ± 0.013 ac	1.731 ± 0.013 c	4.695 ± 0.058 ba	1.203 ± 0.021 a	93.55 ± 1.03 ab
Sal.^®^	9.59 ± 0.07 bc	7.67 ± 0.08 bc	33.88 ± 0.41 a	0.480 ± 0.006 ab	1.728 ± 0.014 c	4.761 ± 0.020 b	1.185 ± 0.013 a	92.46 ± 0.93 a
Sal.^®^ + Cu	9.33 ± 0.12 b	7.46 ± 0.10 b	37.80 ± 0.31 c	0.463 ± 0.006 b	1.689 ± 0.006 b	4.795 ± 0.030 b	1.231 ± 0.017 ab	97.11 ± 1.24 bc
Sal.^®^ + Mo	9.80 ± 0.36 c	7.83 ± 0.29 c	35.56 ± 0.35 b	0.488 ± 0.006 ac	1.655 ± 0.008 a	4.733 ± 0.068 b	1.333 ± 0.024 c	91.52 ± 1.06 a
Sal.^®^ + Fe	10.42 ± 0.15 d	8.24 ± 0.19 d	36.40 ± 0.26 b	0.500 ± 0.004 c	1.655 ± 0.013 a	4.591 ± 0.033 a	1.262 ± 0.020 b	101.20 ± 1.26 c
LSD_0.01_	0.35	0.30	0.90	0.019	0.029	0.113	0.050	4.10
Above-ground parts								
Control	58.73 ± 1.75 a	22.15 ± 0.53 a	5.32 ± 0.12 a	4.067 ± 0.030 a	3.228 ± 0.068 c	2.436 ± 0.030 a	1.204 ± 0.027 a	340.18 ± 3.92 b
Sal.^®^	74.21 ± 0.78 c	31.97 ± 0.36 b	5.88 ± 0.10 b	3.629 ± 0.040 b	2.905 ± 0.076 ab	2.443 ± 0.041 a	1.190 ± 0.041 a	234.52 ± 2.02 a
Sal.^®^ + Cu	73.72 ± 1.81 c	31.01 ± 0.79 b	6.16 ± 0.05 b	3.946 ± 0.036 c	2.882 ± 0.034 a	2.751 ± 0.040 b	1.222 ± 0.009 a	230.56 ± 3.59 a
Sal.^®^ + Mo	71.18 ± 0.15 b	31.62 ± 0.65 b	5.11 ± 0.13 a	4.075 ± 0.072 a	2.916 ± 0.055 ab	2.651 ± 0.057 b	1.231 ± 0.023 a	346.72 ± 7.71 b
Sal.^®^ + Fe	72.36 ± 0.68 bc	31.82 ± 0.54 b	5.88 ± 0.14 b	4.244 ± 0.011 d	3.056 ± 0.051 a	2.717 ± 0.026 b	1.156 ± 0.021 a	429.81 ± 1.60 c
LSD_0.01_	2.17	1.06	0.29	0.110	0.150	0.104	n.s	11.26

Average values ± standard deviations (SDs); DM—dry matter, n.s.—non-significant, Sal.^®^—Salmag^®^; the different letters on the right of the SDs indicate the influence to be significant at *p* ≤ 0.01.

**Table 3:** Effects of innovative materials with chelates on soil properties after harvest of soybean.

Fertiliser	pH	N	Ca	Mg	Cu	Mo	Fe
		Content in g kg^−1^ DM			Content in mg kg^−1^ DM		
Control	5.37 ± 0.04 ab	1.044 ± 0.069 a	4.929 ± 0.139 a	2.143 ± 0.009 a	5.090 ± 0.095 a	1.269 ± 0.015 a	10,251 ± 57 a
Sal.^®^	5.32 ± 0.06 a	1.089 ± 0.038 a	4.680 ± 0.049 a	2.137 ± 0.016 a	5.506 ± 0.059 b	1.325 ± 0.024 b	10,427 ± 81 a
Sal.^®^ + Cu	5.29 ± 0.03 a	1.078 ± 0.077 a	4.927 ± 0.122 a	2.175 ± 0.022 a	5.854 ± 0.047 c	1.272 ± 0.023 ab	10,309 ± 83 a
Sal.^®^ + Mo	5.24 ± 0.02 a	1.078 ± 0.019 a	5.213 ± 0.028 b	2.183 ± 0.044 a	5.057 ± 0.085 a	1.279 ± 0.022 ab	10,308 ± 70 a
Sal.^®^ + Fe	5.45 ± 0.08 b	1.133 ± 0.033 a	4.861 ± 0.101 a	2.128 ± 0.004 a	5.090 ± 0.047 a	1.226 ± 0.014 a	10,317 ± 21 a
LSD_0.01_	0.10	n.s.	0.253	n.s.	0.180	0.052	n.s.

Average values ± standard deviations (SDs); DM—dry matter, n.s.—non-significant, Sal.^®^—Salmag^®^; the different letters on the right of the SDs indicate the influence to be significant at *p* ≤ 0.01.

## Data Availability

The original contributions presented in this study are included in the article. Further inquiries can be directed to the corresponding authors.
